# Pharmacogenomics of Drug Response in Type 2 Diabetes: Toward the Definition of Tailored Therapies?

**DOI:** 10.1155/2015/415149

**Published:** 2015-06-15

**Authors:** Carla Pollastro, Carmela Ziviello, Valerio Costa, Alfredo Ciccodicola

**Affiliations:** ^1^Institute of Genetics and Biophysics “Adriano Buzzati-Traverso”, National Research Council, Via Pietro Castellino 111, 80131 Naples, Italy; ^2^DiST, Department of Science and Technology, “Parthenope” University of Naples, Centro Direzionale, Isola C4, 80143 Naples, Italy

## Abstract

Type 2 diabetes is one of the major causes of mortality with rapidly increasing prevalence. Pharmacological treatment is the first recommended approach after failure in lifestyle changes. However, a significant number of patients shows—or develops along time and disease progression—drug resistance. In addition, not all type 2 diabetic patients have the same responsiveness to drug treatment. Despite the presence of nongenetic factors (hepatic, renal, and intestinal), most of such variability is due to genetic causes. Pharmacogenomics studies have described association between single nucleotide variations and drug resistance, even though there are still conflicting results. To date, the most reliable approach to investigate allelic variants is Next-Generation Sequencing that allows the simultaneous analysis, on a genome-wide scale, of nucleotide variants and gene expression. Here, we review the relationship between drug responsiveness and polymorphisms in genes involved in drug metabolism (*CYP2C9*) and insulin signaling (*ABCC8*, *KCNJ11*, and *PPARG*). We also highlight the advancements in sequencing technologies that to date enable researchers to perform comprehensive pharmacogenomics studies. The identification of allelic variants associated with drug resistance will constitute a solid basis to establish tailored therapeutic approaches in the treatment of type 2 diabetes.

## 1. Introduction

Diabetes is one of the leading causes of mortality in the contemporary society [1]. The last report of the International Diabetes Federation in 2013 indicates an onset rate of about 8.4% in adults and a total number of 382 million cases of diabetes worldwide. This number is estimated to critically rise up to 592 million cases by 2035 [1], so that the World Health Organization (WHO) has defined this phenomenon as a “global outbreak.” There are two more frequent forms of diabetes, both due to defects of insulin action: type 1 diabetes mellitus (T1D), also called “insulin-dependent diabetes” or “juvenile diabetes” [2] and type 2 diabetes mellitus (T2D), also known as “noninsulin-dependent diabetes.” This former is characterized by early onset and occurs because of absolute deficiency of insulin [3], whereas the latter (the most frequent form), with onset in older age, occurs because of an insulin defective function [4]. T2D affects more than 5% of the population of developed countries and its predominance increases worldwide.

Diabetes is a chronic disease, which over time leads to cardiovascular and blood vessels damage and neuro-, nephro-, and retinopathy, with a dramatic impact on health and high costs for all National Health Systems [2].

Intensive programs that consider lifestyle changes to reduce T2D risk have revealed a moderate efficacy in reducing diabetes incidence in at-risk individuals [5]. When lifestyle changes are not sufficient to ameliorate the clinical features of T2D patients, it is necessary to design an appropriate pharmacological approach. In this* scenario*, the pharmacogenomics is a discipline that studies the importance of optimal treatment to patients, starting from the knowledge about the genetic and molecular etiology of the disease. Several studies have shown a widespread variability in glycemic response tolerability, and a plethora of variable effects in patients treated with similar antidiabetic drugs [6, 7]. These lines of evidence represent the starting point of pharmacogenomics [4]. Generally, interindividual variability is mainly determined by single nucleotide polymorphisms (SNPs). Specifically, a relevant fraction of the genetic variability observed in T2D patients has been found in genes directly (or indirectly) related to the activity (or to the metabolism) of oral antidiabetic drugs (OAD). The assumption of these drugs is the first intervention step in T2D management, after the failure of lifestyle changes. Therefore, the identification of genetic variants associated to altered drug responsiveness is a key point in diabetes research, since it is expected to ameliorate the therapeutic approach in a tailored manner. However, other biological nongenetic factors can influence pharmacodynamics of OADs, such as hepatic, renal, and intestinal functions. These considerations highlight the importance of considering both the phenotype (clinical and patho/physiological parameters) and the genotype of T2D patients, in order to choose the most appropriate therapeutic approach [5].

In the last decade, the advent of genome-wide association studies (GWAS) has gradually shifted the genetics of T2D to a step forward, definitely turning pharmacogenetics into pharmacogenomics. Indeed, whereas the former mainly focuses on single drug-gene interaction, the latter faces the relationship among inherited nucleotide variations and drug response, also taking into account gene expression, other genomics features, and epigenetics factors underlying inter- and intraindividual variability [4, 5]. Despite many GWAS have revealed the association among genetic variants and complex traits/diseases, many factors are still underestimated or unexplored, clearly deserving further investigation. For instance, a renewed interest is emerging from the so-called “junk” DNA [8]. Indeed, it is known that the vast majority of nucleotide variants that are associated to complex traits, included T2D, localizes into noncoding regions. Thus, a small fraction of intragenic and intergenic noncoding RNAs (ncRNAs), with still undefined regulatory functions [9, 10], may play a role in the onset and/or progression of multifactorial diseases. Noncoding RNAs levels may also account for the variable drug responsiveness observed in T2D patients.

**Figure 1:**
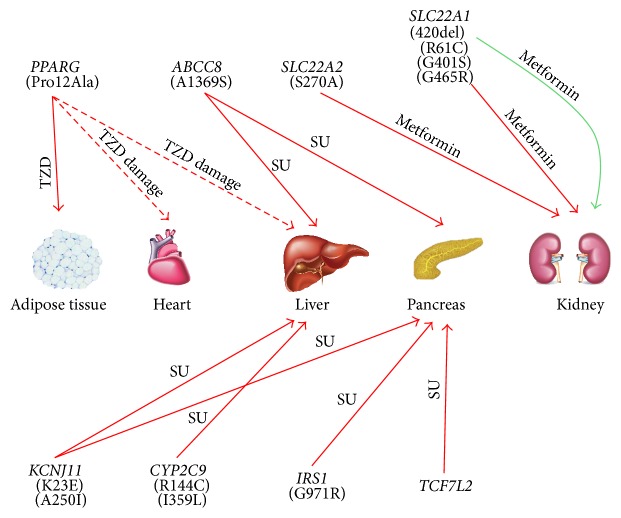
Interactions between gene products and OADs on target organs. Genes and the related “at-risk” SNPs (in brackets) are shown in the upper part. Arrows indicate if a SNP has a negative impact on the responsiveness to a given drug in a specific organ. Red arrows indicate increased drug resistance (or altered drug metabolism), whereas green arrows indicate a beneficial effect of such a SNP. Dashed lines indicate side effects of a given drug. TZD = thiazolidinedione; SU = sulphonylureas; MET = metformin.

In this review, we describe the relationship between drug responsiveness in T2D patients and SNPs, also describing the organs that have a major role in drug metabolism or activity (Figure 1). We also discuss the recent advancements in sequencing technologies, highlighting how they can provide significant contributions to pharmacogenomics studies. The new technological frontiers in the identification of allelic variants associated with altered drug responsiveness will surely constitute a solid basis to design personalized therapeutic approaches in T2D treatment.

## 2. Pharmacogenomics of Antidiabetic Drugs

**Figure 2:**
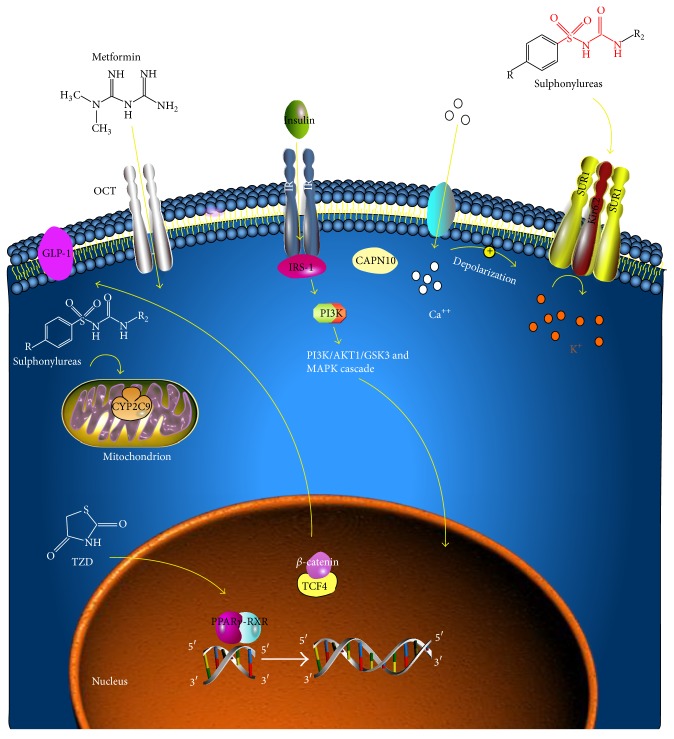
Main proteins involved in uptake and metabolism of OADs. IR = insulin receptor; GLP-1 = glucagon-like peptide-1; SUR1 = sulphonylureas receptor 1; Kir6.2 = potassium inward rectifier 6.2 subunit; PI3K = phosphoinositide 3-kinase; TCF4 = transcription factor 4; RXR = retinoid X receptor; PI3K/AKT1/GSK3 = phosphoinositide 3-kinase/RAC-alpha serine/threonine-protein kinase/glycogen synthase kinase 3; MAPK = mitogen-activated protein kinases.

Currently, the more widely used drugs in T2D treatment are the sulphonylureas, metformin, and thiazolidinediones (troglitazone, pioglitazone, and rosiglitazone).  Figure 2 schematizes the main proteins that are involved in the uptake and metabolism of oral antidiabetic drugs or that are activated upon their administration.

Generally, pharmacogenetics studies consider some clinical endpoints to evaluate drug responsiveness. Among them, the achievement of HbA1c levels <7%, as defined in the guidelines, and an overall reduction in HbA1c represent the most appropriate parameters to consider in T2D pharmacogenetics studies [11]. Another crucial consideration is whether the drug of interest has been used at early or late stages of the disease, where there is a very low probability to reach a significant therapeutic effect.

In Table 1 we summarize a schematic catalogue of SNPs that, according to GWASs, are commonly associated to altered drug responsiveness in T2D. In many cases, these studies have revealed the absence of a significant association among SNPs and the expression levels of the closest gene, showing a wide variability. Such association studies have also underlined the ethnic-specific expression profile of SNPs in tissues crucially involved in glucose homeostasis [12].

## 3. Impact of Polymorphisms in* ABCC8*,* KCNJ11*,* TCF7L2*,* CYP2C9*,* IRS1*, and* CAPN10* Genes on Sulphonylureas Effects

Sulphonylureas (SUs) are widely used drugs in the treatment of T2D. Despite the wide use of these drugs in the clinical practice, different side effects, such as weight gain and increased risk of hypoglycemia, have been frequently [13, 14]. Glibenclamide, gliclazide, glipizide, and glimepiride are the main SUs currently used for T2D treatment [15].

All SUs bind to sulphonylurea receptor 1 (SUR1) and enhance glucose-stimulated insulin release from the pancreatic *β*-cells. Therefore, SUs act by inducing the closure of ATP-sensitive potassium (KATP) channel through the binding with the proteins that form it. Four K^+^ ions are located in the inner pore of KATP channel, whereas outside the channel is formed by four SUR1 molecules [16]. The ATP produced by glucose oxidation in mitochondria causes the closure of KATP channel with the consequent depolarization of *β*-cells membrane, the increased entry of Ca^2+^ ions, followed by the release of presynthesized insulin from *β*-cells. Ultimately, sulphonylureas induce the closure of these channels and the release of insulin through the binding to the specific receptor outside the KATP channel.

Nucleotide variations in genes encoding KATP channel proteins, such as potassium channel inwardly rectifying subfamily J member 11 (*KCNJ11*) and ATP-binding cassette, subfamily C, member 8 (*ABCC8*), are associated with the onset of neonatal diabetes mellitus. Studies on SUs revealed that these drugs might effectively act in response to the defect induced by* KCNJ11* and* ABCC8* mutations in T2D patients [17, 18].* KCNJ11* gene encodes the potassium inward rectifier 6.2 subunit (Kir6.2) of KATP channel, which is implicated in glucose-dependent insulin secretion in pancreatic *β*-cells. GWAs have revealed a strong association between the polymorphism rs5219 in* KCNJ11* (C/T nucleotide substitution that leads to K23E amino acid change) and T2D [19]. Indeed, Javorsky et al. (2012) have demonstrated the impact of K23E amino acid substitution on SUs therapeutic effects in a cohort of 101 Caucasian patients. The study has revealed that “K-allele” homozygous carriers had a higher reduction in HbA1c levels after 6 months of therapy than “EE” carriers (1.04 ± 0.10 versus 0.79 ± 0.12%; *p* = 0.036) [20]. A similar study has been carried out also on Chinese population. In this study, 100 patients were treated for 24 weeks with repaglinide [21]. The authors have reported a significant decrease in HbA1c levels in “EK” and “KK” patients compared to “EE” carries (“EE”: 1.52 ± 1.03%, “EK”: 2.33 ± 1.53%, and “KK”: 2.65 ± 1.73%, *p* = 0.022).

Several studies have reported that sulphonylureas (and also glinides) are able to ameliorate, in T2D patients, the defective insulin secretion. Nonetheless, it has been frequently observed that long-term treatment leads to a progressive decrease in SUs effectiveness. This phenomenon might result from a progressive lack of the insulin-producing capacity of pancreatic *β*-cells. In addition, SUs have proven to be particularly beneficial if combined with metformin, which decreases the extent of insulin resistance [15].

Nucleotide variations in* TCF7L2* gene have been widely associated with T2D onset as well as the effectiveness in SU treatments. Shu et al. (2008) reported that* TCF7L2* is necessary for maintaining the glucose-stimulated insulin secretion (GSIS) and *β*-cell survival. Thus, variations in the level of active* TCF7L2* in *β*-cells may play a crucial role in determining a progressive deficit in the insulin secretion as well as in accelerating T2D progression [22].

T-cell transcription factor 4 (TCF4), the protein encoded by* TCF7L2* gene, is a high mobility group (HMG) box-containing transcription factor, implicated in blood glucose homeostasis. It acts through the binding with *β*-catenin and it mediates Wnt signaling. It is also involved in pancreas development during embryogenesis, and it affects the secretion of glucagon-like peptide 1 (GLP1) by L-cells in the small intestine [23]. Two allelic variants in this gene rs790314 and rs12255372 (C/T and G/T nucleotide variations, resp.) have been associated with T2D. In particular, it has been demonstrated that such variants are the most important predictors of T2D, with a 40% increased risk* per* allele [24, 25]. Genetics of Diabetes Audit and Research Tayside Studies (GoDARTS) has also revealed the relationship between these two allelic variants and therapeutic outcomes in T2D patients treated with sulphonylureas. The GoDARTS study enrolled 901 Scottish T2D patients carrying rs12255372, homozygotes for TT genotype. Patients were treated with sulphonylureas for 3–12 months and compared to individuals with the GG genotype. The results revealed that the TT patients undergoing early SUs treatment had approximately two-fold higher probability to fail (57% versus 17% for TT versus GG resp.) [26]. These results were confirmed by another independent study on 101 Slovakian patients. In this study, T2D patients were supplied six months with SUs. Patients with CT (*n* = 41, HbA1c baseline 8.01 ± 0.13) and TT (*n* = 9, HbA1c baseline 8.06 ± 0.27) genotypes showed a significantly lower reduction of HbA1c levels than CC homozygous patients (*n* = 51, HbA1c baseline 8.06 ± 0.14) [27].

Sulphonylureas are metabolized in the liver by the cytochrome P450 isoenzyme 2C9, encoded by* CYP2C9* gene [28, 29]. Therefore, it is clear that some allelic variants in* CYP2C9* are likely to be associated with T2D susceptibility and/or altered drug responsiveness to SUs. The major risk alleles so far described for this gene are* CYP2C9*∗2 (rs1799853, C/T, Arg144Cys) and* CYP2C9*∗3 (rs1057910, C/T, Ile359Leu) [28].

GoDARTS study has highlighted for the first time also for* CYP2C9* gene the relationship between its variants and the therapeutic response to sulphonylureas. Indeed, treating 1073 T2D patients with SUs the authors observed that 6% of them—carrying two variant alleles (∗2/∗2 or ∗2/∗3 or ∗3/∗3)—had a 0.5% higher reduction in HBA1c levels than ∗1/∗1 homozygous and had 3-4 fold higher probability to reach HbA1c levels <7% [30].

Some studies have also investigated the effects of third generation SUs treatment (combined with metformin) in patients with polymorphisms in* CYP2C9*,* KCNJ11*, and* ABCC8* genes. Klen et al. in 2014 have reported a study on a cohort of 156 Slovenian T2D patients (18–72 years old) treated with SUs monotherapy (*n* = 21) or in combination with metformin (*n* = 135). Glucose levels were monitored (hematic HbA1c) and patients were genotyped for rs1799853 and rs1057910 (∗2 and ∗3 allele, resp.) in* CYP2C9*, for rs5219 and rs5215 in* KCNJ11* and for rs757110 in* ABCC8*. The study revealed that none of these SNPs significantly affected glucose levels. Nonetheless,* CYP2C9*∗3 genotype induced slight hypoglycemic episodes in elderly patients (>60 years old) treated with second-generation SUs more frequently than third generation drugs. Specifically, such difference has been reported for glimepiride treatment used instead of gliclazide, indicating that* CYP2C9* genotypes are relevant to the pharmacokinetics of sulphonylureas [31, 32]. However, the authors could not find any association between hypoglycemic episodes and SNPs in* ABCC8* and* KCNJ11* genes [33].

Additionally, a nucleotide variant (G971A) in* IRS1* (Insulin Receptor Substrate 1) gene has been extensively studied due to its relation with SUs responsiveness. In particular, experimental evidences have shown its association with an increased risk of secondary failure to SUs treatment. The allele frequency of this variant is 2-fold higher in patients with secondary failure to SUs compared to T2D patients that normally respond—in terms of glycemic control—to oral therapy with SUs.* IRS1* gene product acts to stimulate the PI3K/AKT1/GSK3 signaling pathway and in turn glucose transport and glycogen synthesis. The Arg971 polymorphism decreases the phosphorylation of the substrate and allows* IRS1* acting as an inhibitor of PI3K [19, 31, 34].

Finally, there are some SNPs in* CAPN10* gene, such as rs3842570 (intronic indel), rs3792267 (intronic nucleotide change A/G), and rs5030952 (intronic nucleotide change C/T) that are associated to SU responsiveness in T2D patients. However, the exact mechanisms that underlie such phenomenon are still unknown [19].

## 4. Impact of Polymorphisms in* SLC22A1* Gene on Metformin Effects

Metformin is a frequently used drug in the treatment of T2D, as much as SU. It is positively charged at physiological pH, so it turns in hydrophilic changing its pharmacokinetic properties [35]. Metformin is not metabolized in the liver such as sulphonylureas, but it is excreted in the urine. Therefore, the metformin glucose lowering effect is not influenced by genetic variants in genes encoding metabolizing enzymes. Even in this case, pharmacogenetics has taken advantage from GWASs to understand the impact of SNPs in genes encoding metformin transporters on its clinical effects.

Zhou et al. performed a GWAS analyzing about 700 K polymorphisms in 1024 patients treated with metformin, subsequently to GoDARTS [36, 37]. Researchers used GoDARTS and United Kingdom Prospective Diabetes Study (UKPDS) populations for genotyping purposes and obtained the same results [5]. In particular, they demonstrated that nucleotide variations in genes involved in DNA repair and cell cycle control determine altered response to metformin, in terms of glycemic response [37] and considering HbA1c < 7% as treatment achievement. Among them,* SLC22A1* is the most studied gene, as it is involved in the response to metformin. It encodes the organic cation transporter 1 (OCT1). Shu et al. (2008) analyzed the effect of* SLC22A1* gene allelic variants on plasma glucose levels after metformin administration in animal models and healthy volunteers. They identified four polymorphisms in this gene that are associated to T2D susceptibility, that is, R61C, G401S, 420del, and G465R (details about these nucleotide variants are reported in Table 1). Interestingly, they found that glucose lowering was compromised in presence of these SNPs [38, 39].

Moreover, R61C and 420del have been extensively studied since these are the most frequent allelic variants in the Caucasian population. The presence of R61C amino acid change has been demonstrated to determine a reduced expression of OCT1 protein [40].

Interestingly, Christensen et al. studied the effect of eleven polymorphisms in genes encoding other membrane transporters, in association with their effects on plasma glucose levels in 151 T2D patients [41]. Metformin was provided to these patients after insulin treatment. They found that 420del carriers had a more significant decrease in glucose plasma level—after the assumption of metformin—compared to noncarriers [41], indicating a beneficial effect of such SNP on metformin activity.

The frequency occurrence of R61C and 420del in the Asiatic population is lower than in Caucasians, and none of the* SLC22A1* polymorphisms has been associated with a reduced transporter activity [42]. Interestingly, in this population, the genetic variants in* SLC22A2* gene (encoding OCT2) seem to have a stronger association with metformin responsiveness compared to SNPs in* SLC22A1* [5].

## 5. Pro12Ala Polymorphism in* PPARG* and Responsiveness to Thiazolidinediones

The nuclear receptor Peroxisome Proliferator-Activated Receptor (*PPARG*) is a transcription factor that plays a relevant role in glucose and lipid metabolism. It is able to activate the transcription of several metabolic target genes, such as lipoprotein lipase, fatty-acid transcript protein, and aquaporin, which mediate triglyceride hydrolysis, fatty acid, and glycerol uptake [43, 44].* PPARG* is a master gene of adipogenesis, and its functions are very complex due to the huge number of target genes, ligands, and coregulators (coactivators or corepressors) and to the presence of several isoforms, even with opposite or dominant negative activity [44, 45]. Indeed, different studies have revealed the presence of a relevant number of* PPARG* transcripts, strongly suggesting that alternative splicing has an important role in the functioning of such a nuclear receptor [44, 45].

In the last decade,* PPARG* polymorphisms—both in coding and regulatory regions—have been largely analyzed for their possible association to pathologic phenotypes, such as T2D [46–49].

One of the most studied polymorphisms is Pro12Ala (rs1801282), frequently associated with clinical consequences and alterations of the physiological metabolic status [44]. The amino acid modification has been predicted to be responsible of a significant change in the secondary structure of the protein. Thus, it might also affect its functionality [50]. Phenotypically, Pro12Ala has been associated with a decreased risk of T2D, even though conflicting results have been reported in the literature [51, 52]. A study by Hara et al. has shown the association between Pro12Ala and a reduced risk of developing T2D. The authors performed a case-control study on 415 diabetic subjects and 541 nondiabetic subjects in the Japanese population (>60 years old). They revealed that Pro12Ala frequency was significantly lower in the diabetic (0.018, *p* < 0.005) compared to the nondiabetic group (0.043, *p* < 0.005). In detail, T2D individuals carrying Pro/Pro allele were 400 (96.4%), whereas Pro/Ala-Ala/Ala were 15 (3.6%); conversely, nondiabetic patients carrying Pro/Pro allele were 496 (91.7%); instead Pro/Ala-Ala/Ala were 45 (8.3%) [53].

These results are not in agreement with the Finnish Diabetes Prevention Study (FDPS) [54]. Indeed, in this study, 522 individuals with impaired glucose tolerance (IGT) were analyzed after placebo assumption and lifestyle intervention. At the same time, some clinical parameters (weight gain, waist, and hip circumferences, etc.) have been measured in these patients enrolled for the study. A two-fold increase in the risk of developing T2D was reported for Ala carriers in the placebo arm when compared to Pro/Pro homozygous. Moreover, weight gain has been identified as predictor for T2D development, and it has been associated with Pro12Ala SNP Indeed, Ala/Ala homozygous patients were more obese than Pro/Pro homozygous ones. The therapeutic response in presence of Pro12Ala variant has been also evaluated. Such evaluation is crucial to design optimal therapeutic strategies for T2D treatment [55].

A study by Hsieh et al. on 250 diabetic patients (120 men and 130 women) has recently demonstrated the association between Ala allele and a stronger reduction of HbA1c and fasting glucose plasma levels after treatment with thiazolidinediones (TZD; such as pioglitazone, troglitazone, and rosiglitazone). These patients assumed 30 mg/day of pioglitazone for 6 months. One hundred fifty-four patients out of 250 (61.6%) positively responded to the treatment. Levels of HbA1c were 8.56÷1.79 for responders and 8.24÷1.88 for nonresponders (*p* = 0.179) [56]. These findings were further confirmed by a study of Kang et al. (198 diabetic patients) in which 183 T2D patients carried Pro/Pro, 15 carried Pro/Ala, and none of them was Ala/Ala. The diabetic patients carrying Ala12 allele had a higher decrease in fasting glucose plasma levels than Pro/Pro individuals (50.6 ± 27.8 mg/dL versus 24.3 ± 41.9 mg/dL, *p* = 0.026) [57].

Moreover, a study by Blüher and colleagues [58] that enrolled 131 T2D patients revealed no significant differences between Pro/Pro homozygous and Ala carriers in terms of TZD response (defined as HbA1C levels >15% and/or fasting blood glucose decrease >20% after 12 or 26 weeks of treatment with pioglitazone). Furthermore, a larger study, performed on 340 T2D patients, revealed that Pro12Ala is not correlated with any significant difference in troglitazone response [55].

Despite these conflicting results, the association between Pro12Ala polymorphism and TZD responsiveness has to be still investigated at the molecular level. Indeed, the causal relation between this SNP and the altered TZD response has to be functionally proven yet. Notably, TZD have been frequently described to cause significant side effects. Indeed, troglitazone has been recently withdrawn from sale worldwide due to clinical cases of liver damage. Rosiglitazone has been withdrawn from sale in Europe and put under restriction in USA, due to its increased cardiovascular risk associated with its administration [5]. In August 2008, the American Food and Drug Administration recommended monitoring patients under TZDs treatment for increased risk of myocardial ischemia, whose association has been found in several studies [59]. Thus, in light of these considerations, it is particularly relevant to assess, by targeted pharmacogenomics studies, whether TZD side effects may derive from genotype-based differential drug responsiveness. Such consideration holds true also for previously described drugs, commonly used to treat T2D and its complications.

## 6. Next-Generation Sequencing and Diabetes

In the last few years, genotyping and transcriptomics-based studies have gradually shifted from hybridization-based to sequencing-based approaches. Indeed, thanks to the introduction of Next-Generation Sequencing (NGS) technique and of new advanced sequencing platforms, a growing number of studies have shown how polymorphisms can affect gene expression variation among populations [38, 60]. These findings have confirmed that GWAS alone cannot completely capture the complexity of T2D and other multifactorial diseases. Indeed, in the absence of functional studies the potential causative role of SNPs in complex diseases susceptibility is only predictable. Clearly, the combination of different NGS applications (such as RNA-, ChIP-, and DNA-Seq) may help clinicians to dissect the genetic and epigenetic complexity that underlies complex traits/diseases, as well as cancer [61].

Currently, NGS is the most common and powerful approach for genome sequencing, for gene expression studies and to study epigenetic marks. In the last years, this sequencing technology has dramatically reduced the experimental costs, significantly increasing the amount of data output. Among its applications, RNA-Seq has provided a significant improvement in transcriptome analysis, thanks to its ability to detect and quantify low expressed genes, alternative splicing events, posttranscriptional RNA editing, and SNPs expression [60, 62] thanks to the type of sequence (read length), the sequence quality, the high throughput, and its low cost [63].

Notably, as widely discussed in this review, SNPs have been associated with individual pharmacotherapy response. In this light, NGS technology is an optimal candidate to simultaneously explore SNPs and gene expression on a widespread scale. NGS-based studies have been recently performed in animal models to explore the alteration of immunologic and metabolic functions in diabetes. Using RNA-Seq, Kandpal and colleagues investigated the retinal transcriptome of streptozotocin-induced diabetic mice to assess the efficacy of two candidate drugs. Through this approach they found differentially expressed transcripts and quantified the relative abundance of “drug-induced” isoforms after treatment with inhibitors of the advanced glycation end-product receptors and p38 MAP kinase [64]. Similarly, using RNA-Seq to profile human pancreatic islets transcriptome, Eizirik and colleagues revealed that most of candidate gene—identified by GWASs as associated with T1D susceptibility—are expressed in human pancreatic islets and are significantly altered after inflammatory* stimuli* [65]. Due to the higher sensitivity compared to hybridization-based approaches, RNA-Seq has been also used to identify new transcripts potentially implicated in diabetic nephropathy [66].

However, despite the fact that these pioneer studies have started to highlight the potential of NGS for this kind of analyses, to date none of these has still faced the relationship between SNPs and drug responsiveness in T2D patients.

## 7. Conclusions

Since a growing number of studies are pointing out the role of ncRNAs into human diseases, NGS could significantly help researchers to improve the knowledge about SNPs, drug response and the noncoding fraction of the human genome [67, 68].

**Figure 3:**
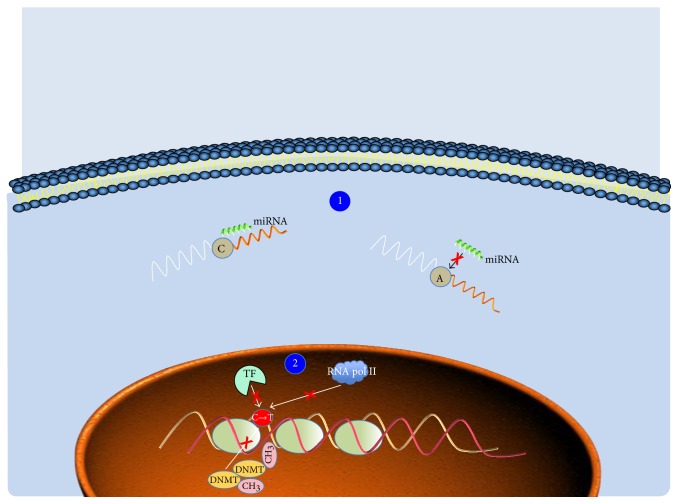
SNPs affecting gene expression and epigenetic mechanism. Schematic representation of how SNPs can potentially affect different processes. (1) A single nucleotide variation (e.g., C to A substitution) may prevent miRNA from binding to its target site in the 3′ untranslated region (UTR) of a gene. (2) SNP may also abrogate the binding site of a transcription factor (TF) and/or the binding of proteins involved in transcription as well as in chromatine remodelling. DNMT = DNA methyltranferase; CH_3_ = methyl group.; RNA pol II = RNA polymerase II.

To the best of our knowledge, any systematic analysis of SNPs in regulatory regions that may affect (abrogate or create) new binding sites for microRNAs (miRNAs) and transcription factors and/or affect nucleotide methylation or chromatin remodeling has not yet been described. In light of this consideration, the usage of NGS to explore this new potential avenue appears crucial (Figure 3).

Overall, we predict that NGS will significantly improve the identification of genetic variants associated with altered drug responsiveness in T2D and that a systematic investigation of how these variations affect gene expression and epigenetic mechanisms is expected to guide better drug use in clinic [62].

## Figures and Tables

**Table 1:** Schematic catalogue of SNPs commonly associated with T2D.

Chr	Position	Localization	Gene	Alleles	SNP ID	Protein change	Association
6	160543148 160560824 160560881 160575837	Exon Exon Exon Exon	*SLC22A1 *	C/T A/G –/A A/C/G	rs12208357 rs34130495 rs35167514 rs34059508	R61C G401S 420del G465R	Metformin metabolism T2D susceptibility

6	160670282	Exon	*SLC22A2 *	G/T	rs316019	S270A	Metformin metabolism T2D development

11	17409572 17408630	Exon Exon	*KCNJ11 *	T/C G/A	rs5219 rs5215	K23E A250I	Sulphonylureas metabolism T2D onset

10	96702047 96741053	Exon Exon	*CYP2C9 *	C/T A/C	rs1799853 rs1057910	R144C I359L	Sulphonylureas metabolism T2D susceptibility

11	17418477	Exon	*ABCC8 *	G/T	rs757110	A1369S	Sulphonylureas metabolism T2D onset

3	12393125	Exon	*PPARG *	C/G	rs1801282	P12A	TZD metabolism T2D onset- development

10	114808902 114758349	Intron Intron	*TCF7L2 *	G/T C/T	rs12255372 rs7903146	— —	Sulphonylureas metabolism T2D development

2	227093745 227660544	Intergene Exon	*IRS1 *	C/T G/A	rs2943641 rs1801278	— G971R	Sulphonylureas treatment T2D onset

2	241534293 241531174 241542703	Intron Intron Intron	*CAPN10 *	Indel A/G C/T	rs3842570 rs3792267 rs5030952	— — —	Probably involved in Sulphonylureas response
